# Lipid Peroxidation Process in Meat and Meat Products: A Comparison Study of Malondialdehyde Determination between Modified 2-Thiobarbituric Acid Spectrophotometric Method and Reverse-Phase High-Performance Liquid Chromatography

**DOI:** 10.3390/molecules22111988

**Published:** 2017-11-16

**Authors:** Anna Reitznerová, Monika Šuleková, Jozef Nagy, Slavomír Marcinčák, Boris Semjon, Milan Čertík, Tatiana Klempová

**Affiliations:** 1 Department of Food Hygiene and Technology, University of Veterinary Medicine and Pharmacy in Košice, 041 81 Košice, Slovakia; anna.reitznerova@student.uvlf.sk (A.R.); jozef.nagy@uvlf.sk (J.N.); slavomir.marcincak@uvlf.sk (S.M.); 2 Department of Chemistry, Biochemistry and Biophysics, University of Veterinary Medicine and Pharmacy in Košice, 041 81 Košice, Slovakia; 3 Institute of Biotechnology, Faculty of Chemical and Food Technology, Slovak University of Technology in Bratislava, 811 07 Bratislava, Slovakia; milan.certik@stuba.sk (M.Č.); tatiana.klempova@stuba.sk (T.K.)

**Keywords:** high-performance liquid chromatography, derivatization, thiobarbituric acid, spectrophotometry, malondialdehyde, lipid oxidation, meat, meat products

## Abstract

The aim of this work was to compare the methods of malondialdehyde detection, as the main secondary product of the lipid peroxidation process, in meat and meat products. Malondialdehyde measurements were performed by two modified methods, the 2-thiobarbituric acid spectrophotometric method and the reverse-phase high-performance liquid chromatography in raw, mechanically-deboned chicken meat and in manufactured frankfurters. The malondialdehyde concentrations measured by the 2-thiobarbituric acid spectrophotometric method were found to be overestimated by more than 25% in raw meat and more than 27% in frankfurters in comparison to the results of reverse-phase high-performance liquid chromatography (*p* < 0.05). The achieved results showed that the presented modified reverse-phase high-performance liquid chromatography method was more applicable and more accurate for the quantification of malondialdehyde in samples of meat and meat products.

## 1. Introduction

Meat is considered to be a very nutritive food and has been rated highly, and associated with good health, by contributing quality protein, B vitamins, iron, and zinc. Meat fat is important in human nutrition with *n*-3 polyunsaturated fatty acid (PUFA) and conjugated linoleic acids (CLAs) playing a beneficial role [1]. Mechanically-deboned meat (MDM), mechanically-recovered meat (MRM), and mechanically-separated meat (MSM) are synonyms used to specify the material obtained by using mechanical force (pressure and/or shear) on animal bones or poultry carcasses from which the bulk of meat has been manually removed [2]. Mechanically-deboned chicken meat (MDCM) is very susceptible to oxidative reactions due to the high lipid content in its composition. These reactions occur from the metabolic transformations of fatty acids in the meat [3]. Mechanical deboning of meat affects the lipid composition of the resulting meat, which normally has a higher lipid content than manually-deboned meats. These extra lipids may originate from subcutaneous fat, the skin, or abdominal fat (depending on the animal species and the method used) but, mainly, it comes from bone marrow and bone tissue [4].

Malondialdehyde (MDA) is one of the most abundant aldehydes generated during secondary lipid oxidation and also probably the most commonly used as an oxidation marker [5]. The presence of oxidized lipids in the diet of humans and animals resulted in an increase of thiobarbituric acid reactive substances (TBARS) in plasma and tissue [1]. MDA is in many instances the most abundant individual aldehyde that results from lipid peroxidation in foods. Its concentration in meat and fish products could reach 300 µM or more [6].

It has also been shown that the initial products of fatty acid oxidation (hydroperoxides) are more toxic to human fibroblasts than the end products MDA or 4-hydroxynonenal [7]. The ability of MDA to alter/cross-link a variety of biological macromolecules may contribute to its toxicity, and its mutagenic/carcinogenic properties could reflect adduct formation with nucleic acid bases. Covalent modification of lipoproteins with MDA may play a pathogenic role in atherosclerosis [8].

Lipid primary oxidation products can generate, if exposed to further oxidation conditions, secondary oxidation products, including aldehydes, ketones, epoxides, hydroxy compounds, oligomers, and polymers. These compounds show a wide variety of physicochemical properties, mainly differing in volatility, polarity, and molecular weight. The most relevant groups of compounds will be commented (aldehydes, volatiles, and polymers), as well as a particular molecule very frequently used as an oxidation marker (malondialdehyde).

Deleterious effects of MDA: induced intracellular oxidative stress, leading to membrane lesions in erythrocytes, and MDA are also genotoxic, reacting with DNA to form highly-mutagenic adducts in human cells [9]. MDA is a highly toxic molecule and is able to disturb many physiological processes in animals and humans. Therefore, MDA should be considered to be more important than a lipid peroxidation byproduct. Moreover, levels of MDA in living organisms have been found to be significantly modified in many pathological situations (e.g., gastric, lung, or breast cancer, and atherosclerotic or cardiovascular diseases) [10]. Among various carbonyl compounds produced as secondary lipid oxidation products, malondialdehyde has received particular attention due to its potential health risk [11], for mutagenic and carcinogenic effects of MDA [12].

A great variety of methodologies have been developed and implemented so far, for determining both primary and secondary oxidation products. The most common methods and classical procedures are described, including peroxide value, TBARS analysis and chromatography [3]. The most widely used method for the determination of MDA is the spectrophotometric determination of pink fluorescent MDA-thiobarbituric acid (MDA-TBA) complex produced after reaction with 2-thiobarbituric acid (TBA) at low pH and high temperature [13,14]. There are several variations of the MDA-TBA method with different conditions of extracting MDA from food samples for example: direct heating of the samples with TBA, sample distillation, lipid extraction with organic solvents, or acid extraction of MDA. In spite of these improving modifications, methods based on the derivatization MDA with TBA are criticized for their lack of sensitivity and their high inaccuracy, since TBA reacts not only with MDA, but also with many other compounds interfering in the TBA assay and resulting in considerable overestimation as well as variability of the results [13,15]. To overcome the biases from the derivatization of MDA with TBA, new derivatising agents were tested. Well-marked improvement of MDA determination accuracy in biological samples were achieved by using 2,4-dinitrophenylhydrazine (DNPH) as a derivative reagent [10,15]. In spite of the stated facts of TBA determination methods, especially spectrophotometric TBA-MDA methods, are still preferable for their simplicity.

The aim of the study was to improve the procedure involving extraction and derivatization of MDA with DNPH and compare the determination methods of malondialdehyde by presenting modified reverse-phase high-performance liquid chromatography (RP-HPLC) and the modified 2-thiobarbituric acid (TBA) spectrophotometric method, and in raw, mechanically-deboned chicken meat and in manufactured frankfurters. The observation of lipid peroxidation process was supplemented by fat content and fatty acid analysis of samples by gas chromatography.

## 2. Results and Discussion

### 2.1. Fatty Acid Composition of Samples

In the presented comparison study of malondialdehyde determination by modified RP-HPLC (MDA-DNPH) and TBA spectrophotometric methods (MDA-TBA) samples of mechanically-deboned chicken meat with high and low pressure, and meat product samples made from these meats were chosen. The examined samples were selected because of the high content of lipids and high content of PUFA, which could result in different MDA concentrations measured by both the TBA and RP-HPLC method. MDCM contains a verifiably higher lipid and PUFA content than manually-deboned meat [2]. Fatty acid (FA) composition in mechanically-deboned chicken meat, and in experimentally-made samples of frankfurters is shown in Table 1.

PUFA is highly susceptible to lipid oxidation [16]. The higher content of PUFA in meat and meat product samples could influence MDA values in samples. PUFA in MDCM samples were around 20%. Low-pressure MDCM contained high concentrations of monounsaturated fatty acid (MUFA) 44.66% and high-pressure MDCM 47.11%. The difference between samples of low- and high-pressure MDCM was observed in parameters of MUFA (*p* < 0.001) and saturated fatty acids (SFA) (*p* < 0.01). In both frankfurter sample groups manufactured from low- and high-pressure MDCM considerably lower PUFA content of 16.82% and 15.38%, respectively, was observed, which was probably caused by the addition of pork back fat in manufactured frankfurter samples.

Total lipids in samples of MDCM were similar—around 16.5%. On the other hand, in manufactured frankfurter samples a noticeably higher content of total lipids was observed (Table 2). For monitoring lipid oxidation processes and MDA concentrations in samples the content of free fatty acids is also important (Table 2). Free fatty acids (FFA) content in MDCM meat samples was lower than in examined frankfurter samples.

### 2.2. Results of Malondialdehyde Determinations

The MDA content analyzed by both the spectrophotometric and HPLC method in raw, mechanically-deboned meat was 0.090, 0.112, 0.071 and 0.090 mg/kg meat, respectively (Table 3). MDA concentrations in meat measured by the TBA method were more than 25% of the HPLC detected values (*p* < 0.05). Similar differences in MDA values between both methods were also observed in tested meat products. The results of the TBA method established overestimating of MDA values by more than 27% in comparison with RP-HPLC method (Table 3).

The higher values obtained with the TBA test have been attributed to several other lipid oxidation products such as alkenals, alkadienals, other aldehydes, and ketones [17,18,19]. The reaction of TBA with these compounds decreases MDA detection specificity and overestimates the results. Because of this distinctive TBA reaction with MDA and the reaction of TBA with other substances, the MDA is not directly determined by TBA spectrophotometric method. The determined products of this reaction are labelled as thiobarbituric reactive substances (TBARS), which was previously reported by authors [20,21,22]. Ross and Smith [23] observed that the TBARS procedure may be used to assess the extent of lipid oxidation in general, rather than to quantify MDA.

**Figure 1:**
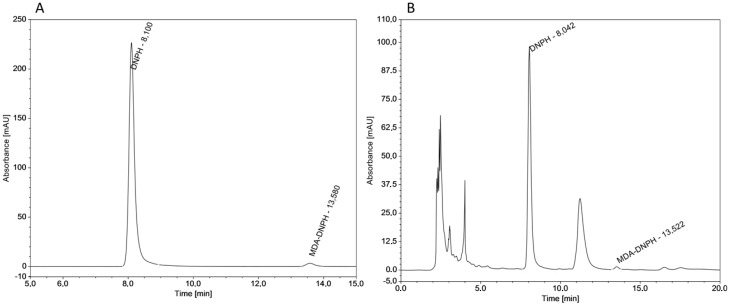
Chromatograms for derivatized MDA-DNPH (**A**) in standard at a concentration of 430 ng/mL and frankfurters (high-pressure MDCM) B (**B**).

On the other hand the HPLC method of MDA determination after derivatization with DNPH does not produce another complex of DNPH with other substances and produces evident peaks, which are easily detectable and separable. In this work the moderately modified HPLC method by Marcinčák et al. [24] was used. Modifying the method by the adjustment of sample quantity (increased to 5 g), decreasing the amount of used solution for the extraction of and increasing the trichloracetic acid (TCA) concentration to 20% (due to effective sample deproteinization and releasing the MDA), we have increased the utilization and reduced the limit of detection in this method. Typical chromatograms at 307 nm for MDA standard and frankfurter samples are shown in Figure 1.

A detailed analysis of chromatograms revealed that the analytical conditions used, the homogenate centrifugation and derivatization yielded clear reaction solutions and, therefore, no further extractions were necessary [10]. The employment of the elution program and a C18-A column with an inner diameter of 5 µm and photodiode detection at the maximum absorbance wavelength of the MDA-DNPH adduct (at 307 nm) allows for very good chromatographic separation of a MDA-DNPH adduct from other endogenous species present in MDCM and frankfurter samples.

We detected MDA content in MDCM samples on 0.07 mg/kg level after low pressure separation and 0.09 mg/kg after high pressure separation using the HPLC method. The results were relatively low, which gave evidence that the material was weakly oxidized. There is not any legislative limit of MDA concentration in meat samples, but MDA over 0.5 mg/kg indicated some oxidation and values above 1.0 mg/kg as possibly unacceptable levels in several studies [25]. Heat treatment (70 °C, 10 min) used in frankfurters processing caused more significantly increased values of MDA of final products than in raw meat used in manufacture (*p* < 0.01; Table 2). The increase of MDA was caused by meat processing and heat treatment. These factors influence the lipid oxidation processes and formation of MDA in meat products [26].

MDA values obtained by the spectrophotometric method were higher in comparison with HPLC method in both MDCM and frankfurter samples. We could conclude that in methods based on MDA-TBA derivatization also formed TBA complexes with other substances, which in spectrophotometric methods overestimated the results. Frankel [26] concluded that the TBA test evidently measures many other decomposition products than malondialdehyde because MDA levels determined in muscle tissues by the TBA test were four to five times higher than HPLC analyses of MDA. These discrepancies may be explained by the reaction of MDA with TBA, which still requires treatment at temperatures of (70–100 °C) for extended incubation times (up to 150 min) in strong acidic conditions (pH 1.5–3.5), which may result in an artifactual peroxidation of sample constituents even in the presence of added antioxidants (i.e., BHT) [15,18]. The results obtained by the modified TBA method overestimated the MDA in MDCM over 25% and in heat treated frankfurter samples over 27% in comparison with the HPLC method. These low differences could be caused by sample preparations, when a low temperature of sample extraction was used. The sample preparation was adapted to eliminate the overestimated results of the final MDA concentration in samples.

**Figure 2:**
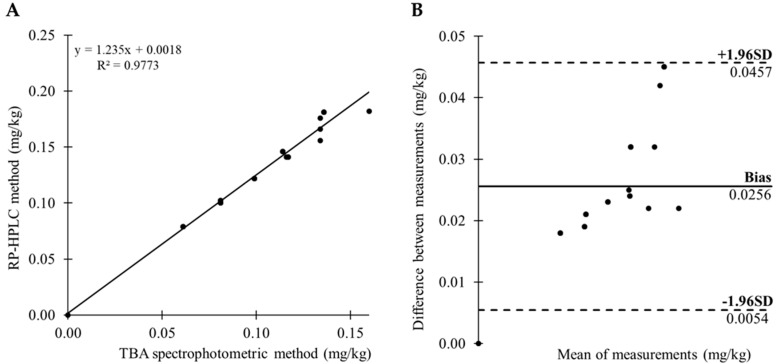
(**A**) Correlation graph between the malondialdehyde determinations by the TBA spectrophotometric and RP-HPLC method; (**B**) The Bland-Altman plot of TBA spectrophotometric and RP-HPLC measurements.

A significant finding of this study is observation of positive correlation between MDA determinations by the TBA spectrophotometric and RP-HPLC methods (*p* < 0.001). The discrepancy between the two used methods is low. The Bland-Altman and regression analysis were performed on the results of MDA determinations by the TBA spectrophotometric and RP-HPLC method. The correlation in MDA values obtained by the TBA spectrophotometric and RP-HPLC method (Figure 2). Scatter plot of the data revealed a linear relationship between the methods (Figure 2A). The bias of two used modified methods was 0.026 ± 0.010 mg/kg (lower limit of agreement = 0.005 and upper limit of agreement = 0.046), what indicated that the spectrophotometric method produces overestimated MDA values compared to the RP-HPLC method (Figure 2B). The 95% confidence interval (CI) for the bias was from 0.021 to 0.030. The 95% CI ranged from −0.002 to 0.013 for the lower limit of agreement, and 0.039 to 0.053 for the upper limit of agreement. The TBA method is not very exact and partially overestimate the results [26], but in our study the results correlated in all samples with the results of the HPLC method. We could conclude that the used modified TBA method is suitable, if we want to analyze the lipid oxidation level. If there is not stated legislative limit for assessment of the maximum MDA amount in meat and meat products, this method is convenient. In the case of the implementation of the MDA limits, it will be necessary to use a specific HPLC method for MDA determination after derivatization with DNPH, which precisely determines MDA in the sample. The specificity of the MDA determination by the presented modified TBA spectrophotometric method in meat and meat product samples could be increased by using the correction factor 0.78, based on the Bland-Altman analysis. The implementation of this correction factor, could significantly prevent the overestimated results of MDA and the results of TBA-MDA spectrophotometric method will be much more accurate.

The MDA concentration depended on lipid content in samples. PUFA perceptiveness for oxidation is well-known [27,28]. The PUFA value in grams is more important than fatty acid profile in meat samples, which was confirmed by the results of our study. The participation of PUFA on fatty acids profile was higher in MDCM samples (average 20%) than in frankfurter samples (average 15%), although the MDA content was higher in frankfurters. MDA in both MDCM and frankfurter samples significantly correlated (*p* < 0.044) with fat content (r = 0.956). The increase of MDA value is correlates with lipid content. Our results agreed with those previously reported by Fuentes et al. [29], who found that lipid oxidation in meat products was significantly affected by lipid content in products. Increasing the lipid content (from 4% to 15%) enhanced lipid oxidation and TBARS increased to a significant extent. MDA values in samples were significantly influenced by free fatty acids (FFA) content. The increase of FFA in samples correlated with an increase of MDA (r = 0.983). The release of FA from triacylglycerols (hydrolysis), is the first step of lipid oxidation, which results in the accumulation of FFA in meat. An important factor in FFA production are the processes in meat products manufacturing (grinding, stirring, separation). Due to this, it is essential to assume higher oxidative damage and higher MDA content in meat products than in unprocessed meat.

We can conclude that the presented modified method for the determination of malondialdehyde by the reverse-phase high-performance liquid chromatography was more precise and accurate than the 2-thiobarbituric acid spectrophotometric method.

The TBA spectrophotometry test overestimates the content of MDA in analyzed samples because of interference with carbohydrates, amino acids, pigments, etc. However, it could be used for the determinations of lipid oxidation processes in meat and meat products during the storage period, different storage conditions, heat treatment, etc.

## 3. Materials and Methods

### 3.1. Fatty Acid Composition

Fatty acids were determined as their methyl esters by gas chromatography according to Čertík et al. [30]. The gas chromatograph (GC-6890 N, Agilent Technologies, Santa Clara, CA, USA) was equipped with a capillary column DB-23 (60 m × 0.25 mm, film thickness 0.25 μm, Agilent Technologies, Santa Clara, CA, USA) and a FID detector (constant flow, hydrogen 35 mL/min, air 350 mL/min, 250 °C). The analysis was performed under temperature gradient (130 °C-1 min; 130–170 °C-6.5 °C/min; 170–215 °C-2.7 °C/min; 215 °C-7 min; 220–240 °C-2 °C/min; 240 °C-2 min) with hydrogen as a carrier gas (flow 2.1 mL/min, velocity 49 cm/s, pressure 174 kPa) and a split ratio of 1/50 (inlets: heater 230 °C, total hydrogen flow 114 mL/min, pressure 174 kPa).

The fatty acid methyl ester peaks were identified by authentic standards for a C4–C24 fatty acid methyl ester mixture (Supelco, Bellefonte, PA, USA) and quantified by an internal standard of heptadecanoic acid (C17:0, Supelco, Bellefonte, PA, USA). The fatty acid concentration was evaluated with ChemStation software B0103 (Agilent Technologies, Santa Clara, CA, USA). All the values were the results of triplicate determination.

### 3.2. Chemicals

All reagents were of analytical grade and purchased from the indicated sources: 35% hydrochloric acid, HCl (Mikrochem, Pezinok, Slovak), trichloroacetic acid, TCA (Fisher Chemical, Loughborough, UK), Ethylenediaminetetraacetic acid disodium salt dihydrate, EDTA (Lach-ner, Neratovice, Czech Republic), 2,6-di-*tert*-butyl-4-methylphenol, BHT and 2-thiobarbituric acid (Sigma-Aldrich, Steinheim, Germany), and *n*-Hexene for spectroscopy (Merck, Darmstadt, Germany). BHT was prepared as a 0.8% (*w*/*v*) solution in hexane. 2,4-Dinitrophenylhydrazine (DNPH, contains min. 30% water) and 1,1,3,3-tetramethoxypropane, (TMP, 99%) were purchased from Acros Organics (Morris Plains, NJ, USA). Analytical grade acetic acid (glacial) was supplied by Merck (Darmstadt, Germany). Gradient-grade acetonitrile for HPLC (Sigma-Aldrich, St. Louis, MO, USA) was used for analysis. Water for chromatography (Merck, Darmstadt, Germany) WAS used for the preparation of a mobile phase and all solutions.

### 3.3. Meat and Meat Product Samples

The meat samples were obtained from VIJOFEL s.r.o., Slovak Republic and HYDINA Slovensko s.r.o., Slovak Republic. Two experimental groups of frankfurters were made from mechanically-deboned chicken meat (MDCM), such as low-pressure MDCM and high-pressure MDCM.

The experimentally made samples of frankfurters were prepared with the following ingredients: 60% MDCM: low-pressure MDCM (group A) or high-pressure MDCM (group B), 30% pork back fat, 20.0 g/kg nitrite salt mixture, 1.0 g/kg polyphosphate, 200.0 g/kg ice, 24.4 g/kg starch, 4.4 g/kg dried garlic, 4.0 g/kg ground black pepper, 3.6 g/kg ground sweet red pepper, 0.2 g/kg ground nutmeg. The MDCM, nitrite salt, polyphosphate and half of the amount of ice was mixed in the meat cutter (MAINCA CM-21, Barcelona, Spain) for 5 min.

Subsequently, other ingredients were added and mixed to a homogeneous mass, until the temperature of 12 °C was reached. The mixture was stuffed into 30 mm diameter hog casings. The frankfurters were heat-treated at 70 °C for 10 min and smoked by cold smoking (EL SPECTRUM, UK 01 EM, Zvolen, Slovak Republic). The experimentally-made samples of frankfurters were packed and stored in a refrigerator at 4 °C.

### 3.4. Determination of Malondialdehyde by the 2-Thiobarbituric Spectrophotometric Method

The extent of lipid oxidation was evaluated as thiobarbituric acid reactive substances (TBARS) by the method of Marcinčák et al. [17] with some modifications. Thiobarbituric acid (TBA) reacted with malondialdehyde, which resulted in a color compound. The thiobarbituric value determination was used to determine the oxidative lipid changes of the samples.

#### 3.4.1. Preparation of Stock Solution and Calibration Curve

MDA was prepared by acid hydrolysis of 1,1,3,3-tetramethoxypropane solution. The amount of 10 µL of tetramethoxypropane (standard solution) was transferred into a 10 mL volumetric flask and 10 mL of 0.1 M HCl was added. The bank was immersed in a water bath for 5 min. Then, the flask was rapidly cooled under running water. Working stock MDA solution was prepared by pipetting 1 mL of the hydrolyzed acetal (solution Y) into a 100 mL homogenous flask and completed with 0.1 mol/L HCl (H_2_O). The MDA stock solution was transferred to a 25 mL volumetric flask and completed with 0.1 M HCl. A MDA working solution at a concentration of 0.1748 μg/mL was used to prepare the calibration curve. Final MDA concentrations for calibration curve were 0.1398, 0.1049, 0.0699, and 0.0346 µg/mL.

Calibration equation for TBA spectrophotometric method was: y = 0.9119x + 0.0067.

#### 3.4.2. Sample Preparation and Measurement

For TBARS determinations 5.00 g of mixed sample in a 50 mL centrifuge tube was weighed. Subsequently 0.5 mL of EDTA was added and stirred, then 2.5 mL of 0.8% BHT, and stirred again. Before homogenization was added 4 mL of 5% TCA was added and homogenized with Ultra-Turrax T18 basic (IKA, Staufen, Germany) at 10,000 rpm for 1 min. After homogenization, the sample was left for 10 min at room temperature and then centrifuged at 4 °C and 3500 rpm for 5 min (Jouan BR4 centrifuge (Jouan Technology for Life, Winchester, VA, USA). Next, the top hexane layer was removed with a pipette and the sample was filtered using filter paper (Whatman No. 4, GE Healthcare, Freiburg, Germany). The filtered sample was transferred to 10 mL volumetric flask and completed with 10% TCA. From the volumetric flask was transferred 4 mL was transferred into the tube and 1 mL of TBA was added, and closed with aluminum foil.

A blank sample was prepared in a separate tube using 4 mL of 10% TCA and 1 mL of TBA. We incubated the tubes in a water bath at 70 °C for 90 min. After that time, the tubes were cooled in an ice bath and after reaching a laboratory temperature the rest of the samples was measured opposite to the blank sample. The calibration curve was set on the UV-VIS spectrophotometer (Helios γ, Thermo spectronic, Cambridge, UK) and TBARS values were measured at 532 nm. The results were quantified as malondialdehyde equivalents (mg/MDA/kg sample).

### 3.5. Determination of Malondialdehyde by Reverse-Phase High-Performance Liquid Chromatography

#### 3.5.1. Standard Preparation

MDA is not commercially available. MDA is an unstable compound and the only possibility to obtain it is by hydrolysis of its stable derivative, bisdiethyl acetal (TMP).

**Figure 3:**
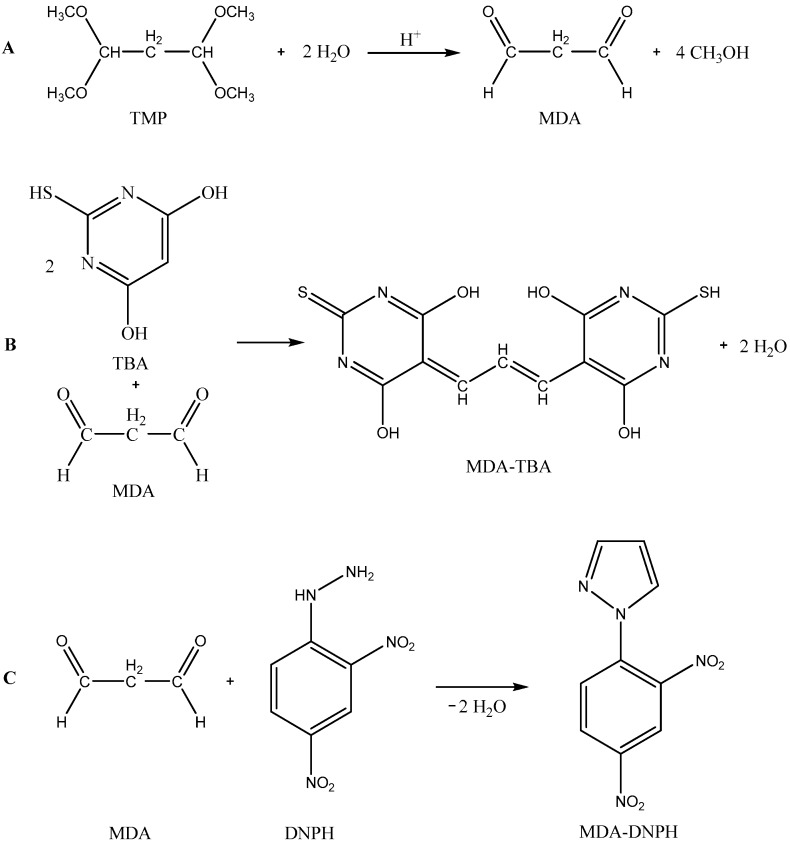
(**A**) The preparation of MDA by acidic hydrolysis of TMP; (**B**) the proposed structure of TBA pigment as a colored adduct between TBA and malondialdehyde, MDA-TBA; and (**C**) the formation of the DNPH derivate of MDA, MDA-DNPH.

MDA standard was prepared as a hydrolyzed product of TMP (Figure 3A). A volume of 10 µL of TMP was diluted in 10 mL of 0.1 M HCl and incubated in a 100 °C water bath for 5 min, then quickly cooled with tap water (solution X). After hydrolysis, a working solution of MDA was prepared by diluting 1.0 mL of obtained solution X with 0.1 M HCl up to 100 mL in a volumetric flask. The resulting MDA standard of 4.37 µg/mL was then diluted with 0.1 M HCl to yield the final concentration of 21.5, 215, 430, 860, 1290 and 1720 ng/mL to obtain the calibration curve. The MDA standard working solution was stored at 5 °C in a dark place and was freshly prepared on a weekly basis. Calibration standards were prepared at the beginning of each analytical run.

#### 3.5.2. Derivatization Reagent Preparation

The DNPH reagent was prepared by dissolving 31 mg of DNPH in 10 mL of 2.0 M HCl and incubated for 30 min at room temperature in the dark. This solution of the derivatising reagent of DNPH was freshly prepared on the day of use. The derivatising procedure for the standards of MDA was the same as for the derivatization of meat sample. A volume of 20.0 µL of derivatized standard solution of MDA-DNPH was injected into the column for HPLC analysis. The formation of the DNPH derivate of MDA, 1-(2,4-dinitrophenyl) pyrazole and its chemical structure are shown in Figure 3.

#### 3.5.3. Sample Preparation

Sample (5.0 g ground meat or meat products) was weighed in a 50 mL centrifuge tube, and 0.5 mL 0.3% EDTA was added immediately. After gently stirring, 2.5 mL 0.8% BHT in hexane was added, and the tube was gently blended again. Before homogenization, 4.0 mL ice-cold 20% TCA was added to the tube and homogenization was performed at 10,000 rpm for 1 min. After the centrifugation (5 min at 3500 rpm, 4 °C), the top hexane layer was removed, and the bottom layer was filtered through Whatman filter paper no. 4. For derivatization, aliquots of the filtrate (500 µL) were transferred into a vial and 50 µL DNPH reagent was added and mixed. Samples were stirred and incubated at room temperature for 30 min in the dark. Finally, sample aliquots (20 µL) of a resulting solution was injected into to column for chromatographic analysis.

#### 3.5.4. Determination of MDA-DNPH by RP-HPLC

Separation and HPLC analysis of MDA as well MDA-DNPH adduct were performed using high-performance liquid chromatography (HPLC). The HPLC system Dionex UltiMate 3000 RS (Thermo Fisher Scientific, Braunschweig, Germany) consisted of a quaternary pump, degasser, automated injector, column oven, and diode array detector (DAD). The DAD detector was set to collect signals within the spectral range of 190–800 nm. Chromatographic separation was achieved on the chromatographic column Polaris C18-A (particle size, 5 μm; column size 250 mm × 4.6 mm; Varian, Santa Clara, CA, USA). Samples were isocratically eluted with a mixture of 0.2% (*v*/*v*) glacial acetic acid in deionized water, and acetonitrile (61:39, *v*/*v*) at a flow rate of 1 mL/min at 25 °C. The injection volume was 20 µL, and the DAD detector was set at 307 nm. Analyses were performed with Chromeleon Chromatography Data System, Version 7.2 (Thermo Fisher Scientific, Braunschweig, Germany) for collecting and processing data. Each analysis was performed in three replicates. All solvents were filtered through a Whatman filter paper no. 4 before use. A calibration curve was prepared mixing a 500.0 µL volume of each of the above mentioned concentrations of standard of MDA and 50 µL of DNPH was added into a vial, and the resulting solution was incubated at room temperature for 30 min in the dark. The clear solution was transferred into a vial and then 20 µL of the resulting solution was injected onto to a column for chromatographic analysis. Triplicate 20 µL injections were made for each standard solution to see the reproducibility of the detector response at each concentration level. The peak area of MDA-DNPH was plotted against the concentration to obtain the calibration graph. The six concentrations of MDA were subjected to regression analysis to calculate the calibration equation and correlation coefficients.

#### 3.5.5. Chromatographic Analysis

The HPLC-DAD system proved to be a good option for the determination of MDA in real meat samples, allowing analysis with good sensitivity and in a total time of 20 min. We used an even higher DNPH solution of 15.6 mM in this study for derivatization to complete the derivatization reaction. The MDA-DNPH peak was identified by the elution profile of the authentic standard. Typical chromatograms for derivatized MDA (MDA-DNPH) in meat products and standard are demonstrated in Figure 3, where it is possible to see that no interferences are present in the region of the retention time of MDA. Peak identification in meat samples was performed by comparison of the retention time. Under the chosen chromatographic conditions, MDA-DNPH showed a retention time of 13.6 ± 0.1 min. For the purpose of peak identification, a 430 ng/mL MDA standard was analyzed and its chromatogram was overlaid with that of the sample chromatograms matched perfectly, indicating that the major peak from MDA was at 13.58 min.

Six calibration standards were analyzed, and their peak areas were plotted against concentration. The equation of linear regression obtained for the six concentration levels, each one injected three times, was: y = 0.0027x − 0.0004, where y is the peak area, and x is the MDA concentration (ng/mL). The regression coefficient *R*^2^ was 0.9979, indicating good linearity. From the analytical curve, the linearity of the method was evaluated, demonstrating a linear interval in the range of 21.5 to 1720 ng/mL.

### 3.6. Statistical Evaluation

Statistical analysis was conducted using the IBM SPSS Statistics version 23 program for Windows (SPSS, Chicago, IL, USA). The MDA content was statistically investigated in samples of meat (low- and high-pressure MDCM) and meat products (frankfurter samples made with low- and high-pressure MDCM during storage). Student’s *t*-test (*p* < 0.05) was used to analyze the results of MDA determinations based on different methods (RP-HPLC and the TBA spectrophotometric method). The Bland-Altman and regression analysis were performed on the results of MDA determinations by two modified methods of the MDA determination. Pearson’s correlation was used to evaluate the relation between MDA determinations, total lipids, and free fatty acid content. It was expressed by the correlation coefficient (r) and significance of the correlations (*p*-value).

## 4. Conclusions

Oxidation of lipids is an important quality indicator of fats, meat, and meat products because oxidized lipids not only change the color, aroma, flavor, texture (sensory properties), and even the nutritive value of the foods, but also generate a lot of harmful biological effects on human health. Products of oxidation are harmful to health due to carcinogenic and atherosclerotic effects, alteration in the composition of cell membranes, or the reduction in high-density lipoproteins.

The results achieved in this study showed that malondialdehyde concentrations measured by the 2-thiobarbituric acid spectrophotometric method were higher than the results of reverse-phase high-performance liquid chromatography. The difference between malondialdehyde concentrations measured by two methods was statistically significant in all samples (*p* < 0.05). However, the results obtained by the TBA spectrophotometric method, which is not very exact and partially overestimates the results, correlated with the results of the HPLC method. For this reason, we can conclude that the presented modified TBA spectrophotometric method is suitable for monitoring the lipid oxidation processes in meat and meat samples. The presented modified determination methods showed that reverse-phase high-performance liquid chromatography was more applicable and accurate for the quantification of secondary lipid oxidation product, malondialdehyde, in meat and meat products.

## Figures and Tables

**Table 1:** Fatty acid composition (% of total fatty acid) in raw materials for meat production (low- and high-pressure MDCM and meat products—frankfurters (means ± SD).

	Mechanically Deboned Chicken Meat			Frankfurter Samples		
Fatty Acid (% of Total Fatty Acid)	Low Pressure	High Pressure	*p*-Value	Low Pressure Deboned	High Pressure Deboned	*p*-Value
C12:0	4.36 ± 0.49	1.36 ± 0.22	**<0.001**	0.74 ± 0.04	0.32 ± 0.00	**<0.001**
C14:0	1.82 ± 0.08	1.09 ± 0.07	**<0.001**	1.49 ± 0.03	1.39 ± 0.01	**<0.001**
C16:0	22.53 ± 0.60	23.56 ± 0.10	**0.004**	21.80 ± 0.32	23.00 ± 0.11	**<0.001**
C16:1–7c	4.95 ± 0.45	6.08 ± 0.12	**<0.001**	2.69 ± 0.05	2.77 ± 0.03	**0.013**
C18:0	6.92 ± 0.44	6.68 ± 0.16	0.290	11.03 ± 0.12	11.72 ± 0.05	**<0.001**
C18:1–9c	36.11 ± 0.40	37.35 ± 0.26	**<0.001**	40.82 ± 0.11	41.14 ± 0.33	0.069
C18:1–11c	2.72 ± 0.12	2.73 ± 0.02	0.815	3.09 ± 0.09	2.78 ± 0.25	**0.026**
C18:2–9c.12c	15.63 ± 0.62	16.47 ± 0.20	**0.016**	14.03 ± 0.08	12.90 ± 0.07	**<0.001**
C18:3–6c.9c.12c	0.16 ± 0.00	0.16 ± 0.01	0.813	0.05 ± 0.00	0.05 ± 0.00	0.013
C18:3–9c.12c.15c	1.20 ± 0.03	1.17 ± 0.03	0.188	1.03 ± 0.01	0.97 ± 0.01	**<0.001**
C20:0	0.08 ± 0.01	0.08 ± 0.01	0.183	0.22 ± 0.01	0.21 ± 0.01	**0.049**
C20:1–11c	0.40 ± 0.02	0.42 ± 0.00	0.051	0.86 ± 0.01	0.88 ± 0.01	**0.001**
C20:2–11c.14c	0.23 ± 0.05	0.22 ± 0.01	0.695	0.54 ± 0.01	0.52 ± 0.01	**<0.001**
C20:3–8c.11c.14c	0.32 ± 0.09	0.25 ± 0.03	0.119	0.15 ± 0.01	0.12 ± 0.00	**<0.001**
C20:4–5c.8c.11c.14c	1.39 ± 0.44	1.27 ± 0.20	0.596	0.56 ± 0.07	0.42 ± 0.02	**0.001**
C20:3–11c.14c.17c	0.04 ± 0.01	0.03 ± 0.00	0.073	0.14 ± 0.00	0.14 ± 0.00	1.000
C22:0	0.04 ± 0.01	0.04 ± 0.01	0.646	0.02 ± 0.00	0.02 ± 0.00	**0.009**
C20:5–5c.8c.11c.14c.17c	0.11 ± 0.03	0.08 ± 0.01	**0.040**	0.04 ± 0.01	0.02 ± 0.00	**<0.001**
C22:5–7c.10c.13c.16c.19c	0.33 ± 0.09	0.25 ± 0.05	0.113	0.18 ± 0.02	0.16 ± 0.01	0.072
C22:6–4c.7c.10c.13c.16c.19c	0.21 ± 0.07	0.18 ± 0.03	0.397	0.10 ± 0.02	0.08 ± 0.01	0.216
Ʃ SFA	35.71 ± 1.63	32.77 ± 0.55	**0.003**	35.27 ± 0.52	36.64 ± 0.18	**<0.001**
Ʃ MUFA	44.66 ± 1.00	47.11 ± 0.42	**<0.001**	47.89 ± 0.27	47.97 ± 0.62	0.809
Ʃ PUFA	19.61 ± 1.43	20.08 ± 0.56	0.507	16.82 ± 0.22	15.38 ± 0.13	**<0.001**
Ʃ PUFA*n*-3	1.52 ± 0.13	1.43 ± 0.07	0.204	1.16 ± 0.03	1.08 ± 0.02	**0.001**
Ʃ PUFA*n*-6	17.02 ± 1.07	17.74 ± 0.40	0.158	14.60 ± 0.15	13.32 ± 0.09	**<0.001**

*p*-values with statistically significant differences were highlighted in bold.

**Table 2:** Total lipids and free fatty acids content in samples (means ± SD).

Parameters	Mechanically Deboned Chicken Meat			Frankfurter Samples		
	Low Pressure	High Pressure	*p*-Value	Low Pressure	High Pressure	*p*-Value
Total lipids (%)	16.08 ± 0.26	16.65 ± 0.28	**0.008**	38.07 ± 0.68	42.73 ± 0.57	**<0.001**
FFA (%)	8.58 ± 0.59	12.52 ± 1.32	**<0.001**	18.42 ± 1.46	16.34 ± 0.76	**<0.001**

FFA: free fatty acids; *p*-values with statistically significant differences were highlighted in bold.

**Table 3:** Malondialdehyde concentration in experimental samples determined by modified TBA spectrophotometric method and RP-HPLC (means ± SD).

Samples	Methods of MDA Determination (mg/kg)		*p*-Value
	Spectrophotometric Method	RP-HPLC	
Raw material for meat production:			
Low-pressure MDCM	0.090 ± 0.012	0.071 ± 0.011	**0.017**
High-pressure MDCM	0.112 ± 0.011	0.090 ± 0.010	**0.004**
Frankfurters:			
Low-pressure MDCM	0.161 ± 0.022	0.126 ± 0.015	**0.006**
High-pressure MDCM	0.156 ± 0.013	0.124 ± 0.011	**0.001**

MDCM: mechanically deboned chicken meat; *p*-values with statistically significant differences were highlighted in bold.
